# Comparison of Bioelectrical Impedance Analysis (BIA)-Derived Parameters in Healthy Volunteers and Critically Ill Patients

**DOI:** 10.3390/life14010027

**Published:** 2023-12-23

**Authors:** Robbert Cleymaet, Marvin D’Hondt, Thomas Scheinok, Luca Malbrain, Inneke De Laet, Karen Schoonheydt, Hilde Dits, Niels Van Regenmortel, Michael Mekeirele, Colin Cordemans, Andrea Minini, Paolo Severgnini, Wojciech Dabrowski, Adrian Wong, Manu L. N. G. Malbrain

**Affiliations:** 1 Department of Oromaxillofacial and Plastic Surgery, Ghent University Hospital, 9000 Ghent, Belgium; 2 Department of Neurology, University Hospital Brussels (UZB), 1090 Jette, Belgium; 3 University School of Medicine, Katholieke Universiteit Leuven (KUL), 3000 Leuven, Belgium; 4 Department of Intensive Care Medicine, Ziekenhuis Netwerk Antwerpen, ZNA Stuivenberg, 2000 Antwerp, Belgium; 5 Department of Intensive Care Medicine, University Hospital Brussels (UZB), 1090 Jette, Belgium; 6 Faculty of Medicine and Pharmacy, Vrije Universiteit Brussel (VUB), 1090 Jette, Belgium; 7 Department of Intensive Care, AZ Sint-Maria Hospital, 1500 Halle, Belgium; colincordemans@gmail.com; 8 School of Anaesthesia and Intensive Care, Dipartimento di Biotecnologie e Scienze della Vita, Insubria University, 21100 Varese, Italy; paolo.severgnini@uninsubria.it; 9 Department of Anestesia e Rianimazione Cardiologica, ASST dei Sette Laghi, Ospedale di Circolo e Fondazione Macchi, 21100 Varese, Italy; 10 First Department of Anaesthesiology and Intensive Therapy, Medical University Lublin, 20-954 Lublin, Poland; 11 Department of Intensive Care Medicine and Anaesthesia, King’s College Hospital, Denmark Hill, London SE5 9RS, UK; 12 International Fluid Academy, 3360 Lovenjoel, Belgium; 13 Medaman, Medical Data Management, 2440 Geel, Belgium

**Keywords:** bioelectrical impedance analysis (BIA), ICU, critically ill, healthy volunteers, prognostic value, mortality, capillary leak, body water, extracellular water, intracellular water, abdominal pressure, abdominal hypertension, fluid overload, fluid accumulation, body composition, fluid composition, malnutrition

## Abstract

Objective: To compare bioelectrical impedance analysis (BIA)-derived parameters in healthy volunteers and critically ill patients and to assess its prognostic value in an ICU patient cohort. Design: Retrospective, observational data analysis. Setting: Single centre, tertiary-level ICU (Ziekenhuis Netwerk Antwerpen, ZNA Stuivenberg Hospital). Patients: 101 patients and 101 healthy subjects, participants of International Fluid Academy Days. Measurements and main results: Compared to healthy volunteers, both male and female ICU patients had significantly higher values for total body water (TBW), extracellular water (ECW), extracellular fluid (ECF), plasma, and interstitial fluid volumes. The phase angle was significantly lower and the malnutrition index was significantly higher in ICU patients, regardless of gender. Non-survivors in the ICU had significantly higher extracellular water content (ECW, 50.7 ± 5.1 vs. 48.9 ± 4.3%, *p* = 0.047) and accordingly significantly lower intracellular water (ICW, 49.2 ± 5.1 vs. 51.1 ± 4.3%, *p* = 0.047). The malnutrition index was also significantly higher in non-survivors compared to survivors (0.94 ± 0.17 vs. 0.87 ± 0.16, *p* = 0.048), as was the capillary leak index (ECW/ICW). Conclusions: Compared to healthy volunteers, this study observed a higher malnutrition index and TBW in ICU patients with an accumulation of fluids in the extracellular compartment. ICU non-survivors showed similar results, indicating that ICU patients and a fortiori non-survivors are generally overhydrated, with increased TBW and ECW, and more undernourished, as indicated by a higher malnutrition index.

## 1. Introduction

Intravenous fluid therapy is one of the cornerstones in the management of critically ill patients [1]. However, little is known about their body fluid compositions. Aggressive fluid resuscitation can lead to fluid overload (FO) through an increase in body fluid in all compartments (extracellular water (ECW), intracellular water (ICW), and total body water (TBW = ICW + ECW)) due to vascular endothelial hyperpermeability and subsequent capillary leaks [2,3]. Fluid overload has been associated with adverse outcomes and mortality and thus needs to be avoided [4,5,6,7,8].

FO is traditionally defined as a 10% increase in the cumulative fluid balance from baseline body weight, and various methods exist to assess it [9,10,11]. A major challenge is obtaining accurate bedside information in critically ill patients concerning their fluid status, and confusion around the various terms used has further compounded the problem of consistent application. The term ‘fluid overload’ describes an all or nothing phenomenon; thus, recent studies suggest the use of the term ‘fluid accumulation syndrome’ (FAS) instead, defined as any percentage of fluid accumulation (FA) associated with new-onset organ dysfunction or failure [12].

Reference methods for the measurement of TBW are isotope techniques such as dual energy X-ray absorptiometry (DXA), deuterium dilution, and, more recently, a technique utilizing radioactively labelled albumin. However, these isotope techniques are impractical at the bedside in an ICU setting [13]. Other methods include the calculation of intravascular filling pressures (e.g., central venous pressure), the assessment of daily and cumulative fluid balances, and the measurement of body weight. None of these techniques have proven to be fully reliable due to a lack of sensitivity and an inability to differentiate between the various fluid compartments [7,14,15,16,17]. 

Bioelectrical impedance analysis (BIA) parameters have been proposed as a safe, fast, and non-invasive bedside alternative to evaluate whole body and fluid compartment compositions [18]. Alternatively, bioimpedance spectroscopy (BIS) could be more accurate, although further research is needed on the subject [19]. BIA measures electrical impedance by passing a small single- or multiple-frequency (SF-BIA or MF-BIA) alternating current (1–10 microA) through the body. Through raw electrical measures (e.g., impedance, capacitance, reactance) further parameters including TBW, ICW, ECW, and volume excess (VE) can be computed [20,21].

Recent studies show that parameters obtained by BIA measurements can be used to assess body fluid composition and nutritional status, but BIA’s accuracy and interpretation in critically ill patients are still a matter of debate and further research is necessary [19,22]. Hence, the aim of our study was to compare BIA-derived variables in healthy volunteers and critically ill patients. In addition, we assessed its value in differentiating survivors from non-survivors in the ICU population.

## 2. Materials and Methods

### 2.1. Ethical Regulations

The study was conducted in accordance with the study protocol, the Declaration of Helsinki, and applicable regulatory requirements. The local Institutional Review Board and Ethics Committee of the Ziekenhuis Netwerk Antwerpen (ZNA) Stuivenberg approved the protocol (EC approval number: 4737 with insurance policy Ethias 45.313.314). The analysis and results on the prognostic value of the presence of fluid overload in the 101 ICU patients has been published previously under the Open Access CC BY Licence 4.0 [7]. In view of the retrospective and observational nature of the study, which did not require a deviation from standard clinical ICU practice, informed consent from the patient or the next of kin was not essential. 

### 2.2. Study Design

The hypothesis of the study is that BIA-derived parameters are a useful tool to assess fluid compartment status and are of prognostic value in critically ill patients. The study was an unmatched, observational cohort study using convenience sampling of ICU patients. Specific fluid status data collection was performed at one given time point. To compare the fluid status data of the patients, the control group consisted of randomly selected healthy volunteers who participated at the International Fluid Academy Days, held in Antwerp Belgium at the Hilton Old Town Congress Centre between 2013 and 2017.

As this was an observational hypothesis-generating pilot study, no formal sample size nor power calculations were performed. Data on BIA-derived parameters were available concerning 101 ICU patients during the study period (from February 2013 until July 2015) and therefore a same sample size of 101 healthy volunteers was used.

### 2.3. Data Collection

Excel 2019 (Microsoft, Redmond, WA, USA) was used to create an anonymized database for each patient and volunteer. For both groups, baseline demographic data, including the age, gender, and body mass index (BMI), were collected.

For the patient cohort, additional data was recorded, such as ICU and hospital admission and discharge dates as well as severity scores such as the Acute Physiology and Chronic Health Assessment (APACHE-II), Simplified Acute Physiology Score (SAPS-II), and Sequential Organ Failure Assessment (SOFA) scores. The cumulative fluid balance was determined by calculating the difference between daily inputs and outputs from the time of admission until the date of BIA measurement. If available, advanced heamodynamic parameters were recorded such as intra-abdominal pressure (IAP) (obtained via an indwelling bladder catheter (FoleyManometer, Holtech Medical, Charlottenund, Denmark)) and transpulmonary thermodilution (PICCO, Maquet Getinge, Sölna, Sweden)). Lastly, various laboratory results were retrieved from the medical records, including hematocrit (HCT, %), total protein (g/L), albumin (g/L), C-reactive protein (CRP, mg/dL), urea (mg/dL), measured and calculated osmolality (mmol/L), glucose (mg/dL), sodium (Na, mmol/L), potassium (K, mmol/L), and creatinine (mg/dL) levels. 

### 2.4. BIA Measurements

BIA measurements were performed using a BioScan 920-II multi-frequency analyzer (Maltron International, Essex, UK) as per the manufacturer’s instructions. Two electrodes were placed on the wrist and two on the ankle and bioelectrical impedance was measured at four frequencies (5, 50, 100, and 200 kHz) in a completely supine position. The 50 kHz frequency was used as the standard reference frequency because resistance and reactance (and thus impedance) are best measured at this frequency and because most reference data is available at this frequency [22]. Additionally, the cell’s property as a capacitator is only present at low frequencies, which explains why 50 kHz measures only ECW while high frequencies measure TBW [23]. The device has been validated and tested previously and showed good reproducibility in healthy volunteers [24]. The following derived parameters were obtained for both the patient cohort and the healthy volunteers (for the patient cohort this was performed during the first week of their stay (on average, on day 5.1 ± 2)):

– TBW, ICW, and ECW (liter and %)
– The ECW/ICW ratio
– Volume excess (VE, L)
– Fat-free mass (FFM, kg and %), fat-free mass hydration (FFMH, %), and fat mass (FM, kg and %)
– Protein mass (kg), mineral mass (kg), bone mass (kg), muscle mass (kg)
– Resting metabolic rate (RMR), glycogen deposits (g)
– Total body calcium (TBCa, g)
– Malnutrition index.

Algorithms to calculate these derived BIA parameters from the raw data are property of the company and not at disposal for the public. The analysis of the presence and prognostic impact of FO has been previously reported, and the FO percentage was defined as the percent increase in VE divided by initial or dry body weight [7]. 

### 2.5. Statistical Analysis

Continuous variables were tested for normality via the Shapiro–Wilk test. Normal distributed variables were expressed as mean ± standard deviation, while those characterized by a non-normal distribution were given as median (with interquartile ranges). Statistical differences in or between the patient cohort and healthy volunteers were determined by the two-sided unpaired Student’s *t*-test for continuous variables and the Chi-square test for categorical variables. A receiver operating characteristic (ROC) curve analysis was performed to identify the thresholds for the malnutrition index and ECW/ICW ratio with the best sensitivity and specificity to predict outcome. A post hoc analysis was performed in relation to the presence of malnutrition and capillary leak based on these best thresholds. A Kaplan–Meier survival curve was created for the patients with and without malnutrition and those with and without capillary leak. Stepwise multiple logistic regression analysis was performed previously to determine independent predictors for ICU mortality [7]. A two-sided *p*-value < 0.05 was considered significant.

## 3. Results

### 3.1. ICU Patients vs. Healthy Volunteers

In terms of patient demographics, the male to female ratio was significantly different between healthy volunteers (65% male) and ICU patients (35% male) (*p* = 0.010). Therefore, females (*n* = 103) and males (*n* = 99) were analysed separately to compare ICU patients to healthy volunteers. The female ICU patients (*n* = 35) were significantly shorter (*p* < 0.001), were heavier (weight (*p* = 0.005), had higher BMI (*p* < 0.001)), and were older (*p* < 0.001) than the healthy female volunteers (*n* = 68). Male ICU patients (*n* = 66) were only significantly shorter (*p* < 0.001) and older than the healthy male volunteers (*n* = 33) (*p* < 0.001) (Table 1).

Regarding raw BIA values, both the female and male ICU patients had significantly lower impedance (*p* < 0.001), phase angle (female *p* < 0.001, male *p* = 0.038), resistance (*p* < 0.00 1), and reactance (*p* < 0.001) values but a higher capacitance at 50 kHz compared to healthy individuals (*p* < 0.001) (Table 1).

Female ICU patients had significantly higher VE, absolute TBW (L), ECW, ECW/ICW ratio, extracellular fluid (ECF), plasma fluid, interstitial fluid, and FFMH (%) values compared to healthy women (Table 2). In contrast, healthy women had a significantly higher relative ICW (%) and body density (Table 2). There was no significant difference in dry weight, body density, or extracellular solids (ECS) between the two groups. Male ICU patients had significantly higher VE, TBW, ECW, ECW/ICW, ECF, plasma fluid, interstitial fluid, and FFMH (%) values (Table 2). In contrast, healthy men had a significantly higher relative ICW (%) and ECS (Table 2). There was no significant difference in dry weight, body density, or absolute ICW between the two groups. All results are listed in Table 2. 

Lastly, nutritional status in healthy volunteers and ICU patients was assessed for both men and women, including phase angle, malnutrition index, resting metabolic rate (RMR), fat-free mass (FFM), body cell mass (BCM), extracellular mass (ECM), fat mass, protein, mineral, muscle, total body potassium (TBK), total body calcium (TBCa), and glycogen levels (Table 3). Women in the ICU have higher malnutrition index, absolute and relative fat, and ECM values but do have a lower RMR and relative FFM than healthy women (Table 3). Men in the ICU have higher malnutrition index and ECM values but lower RMR, BCM, protein, mineral, muscle, TBK, TBCa, and glycogen levels than healthy men (Table 3). All other findings were not found to be statistically different between the two groups. All results on nutritional status are listed in Table 3.

The tabular results of the comparison for the whole group of healthy volunteers compared to ICU patients, including the raw data at other frequencies, is provided in ESM Tables S1–S4. ESM Figure S1 shows boxplots comparing body water distribution in female and male patients, and ESM Table S5 compares demographic and bioelectrical impedance analysis (BIA) variables between female and male volunteers and patients.

**Figure 1:**
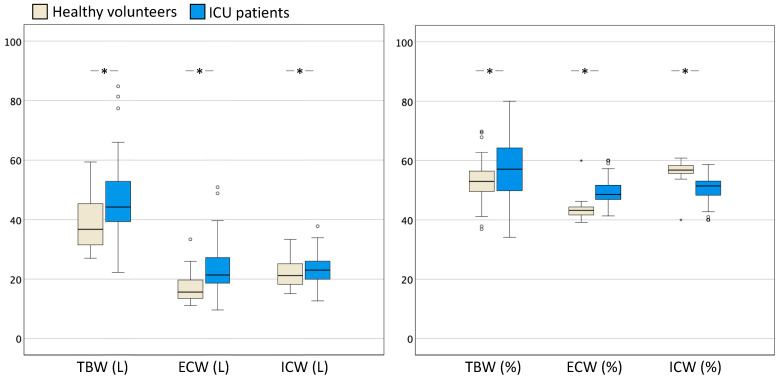
Boxplots comparing body water distribution healthy volunteers (*n* = 101) and a group of ICU patients (*n* = 101). Box and whisker plots comparing body water (TBW, ECW, and ICW) distribution between the two groups. The left panel shows absolute values in litres (L) and the right panel shows relative values in terms of the percentage (%) of TBW. The error bars are the 95% confidence interval, the bottom and top of the box are the 25th and 75th percentiles, the line inside the box is the 50th percentile (median), and any outliers are shown as open circles. A *p* value < 0.05 between groups is indicated with *. ECW = extracellular water; ICW = intracellular water; ICU = intensive care unit; TBW = total body water.

Figure 1 illustrates the differences in absolute (expressed in L) and relative (%) body composition in healthy individuals compared to critically ill patients in the ICU. 

**Figure 2:**
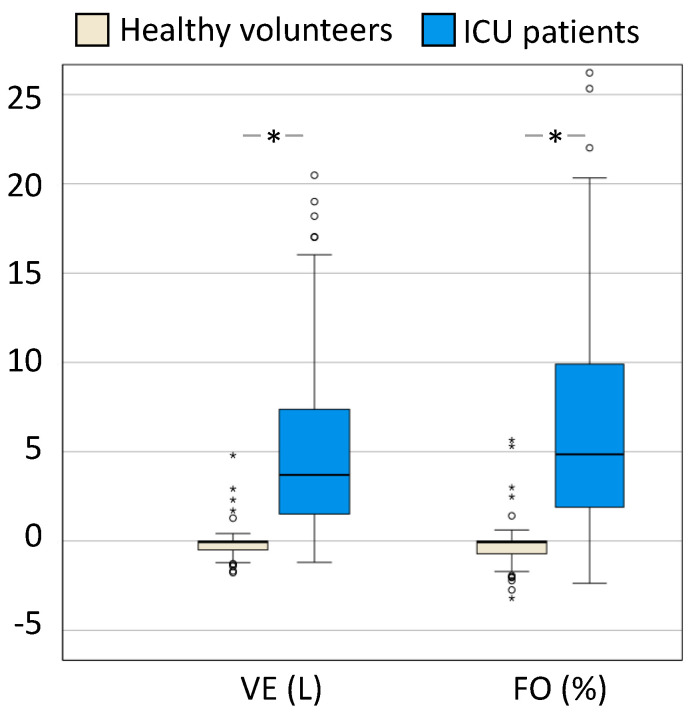
Boxplots comparing volume excess and fluid overload. Box and whisker plots comparing volume excess (VE) in L and (FO) expressed as a percentage in relation to body weight in ICU patients (*n* = 101) and healthy volunteers (*n* = 101). The error bars are the 95% confidence interval, the bottom and top of the box are the 25th and 75th percentiles, the line inside the box is the 50th percentile (median), and any outliers are shown as open circles. A *p* value < 0.05 between groups is indicated with *.

Figure 2 illustrates the differences in volume excess (L) and fluid overload or accumulation (%) in healthy volunteers compared to critically ill patients.

### 3.2. Survivors vs. Non-Survivors in the ICU Population

Table 4 presents the results of the various demographic and clinical variables of 101 patients who were hospitalized for various illnesses. The patients were divided into two groups: those who survived in the ICU (*n* = 61) and those who died (*n* = 40), with an overall ICU mortality of 39.6%. The hospital mortality was 47.5% (*n* = 48). 

When dichotomized in groups, the ICU and hospital mortality were lower in the malnutrition (*n* = 66) and capillary leak (*n* = 76) group compared to patients without these conditions. 

**Figure 3:**
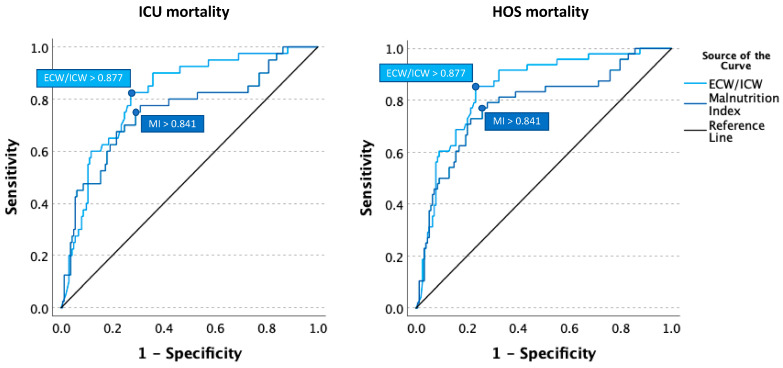
Receiver operating characteristic (ROC) curves assessing the predictive values the for malnutrition index (MI) and the capillary leak index (calculated by the ECW over ICW ratio) in relation to ICU (**left panel**) and hospital (HOS) mortality (**right panel**).

Based on the results of area under the ROC analysis for the total population, the best thresholds for ICU mortality prediction were 0.877 for capillary leak (with a sensitivity and specificity of 82.5% and 72.8%, respectively) and 0.841 for malnutrition (with a sensitivity and specificity of 75% and 71%, respectively). Figure 3 shows the ROC curves. 

**Figure 4:**
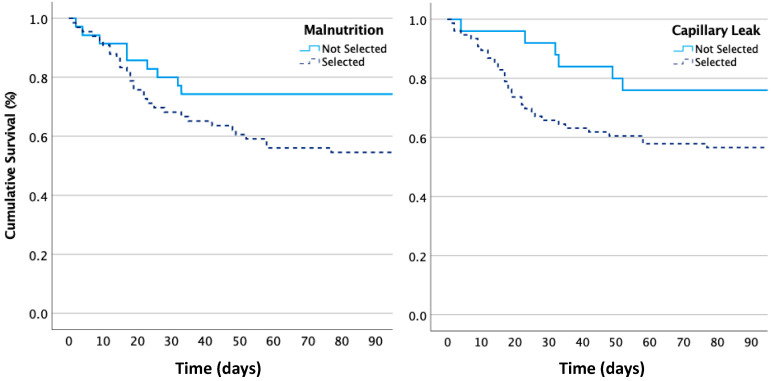
Kaplan–Meier ICU survival curve in patients, dichotomised by the presence or absence of malnutrition (**left panel**), defined as a malnutrition index > 0.841, and by the presence or absence of capillary leak (**right panel**), defined as an ECW/ICW ratio > 0.877. The overall ICU mortality was 39.6% (*n* = 40) for patients in this study. The log rank test showed that the survival curves for patients with malnutrition or capillary leak were significantly lower than those without these conditions.

The results of the different parameters with respect to hospital mortality are listed in ESM Tables S6 and S7. The ICU mortality was 45.5% (*n* = 30) in patients with malnutrition vs. 28.6% (*n* = 10) in those without (*p* = NS). The ICU mortality was 43.5% (*n* = 30) in patients with capillary leak vs. 28% (*n* = 7) in those without (*p* = 0.07). The hospital mortality was significantly higher, at 56% (*n* = 37), in patients with malnutrition vs. 31.4% (*n* = 11) in those without (*p* = 0.015). A similar trend in hospital mortality, at 53.9% (*n* = 41), was observed in patients with capillary leak vs. 28% (*n* = 7) in those without (*p* = 0.021). The Kaplan–Meier log rank (Mantel–Cox) was not significant for ICU mortality but did show a significant difference in hospital mortality for the presence of malnutrition (*p* = 0.029) and capillary leak (*p* = 0.026). Figure 4 shows the Kaplan–Meier survival curves.

Several other variables were found to be significantly different between the two groups. Non-survivors were significantly older (*p* = 0.007), had shorter hospital stays (*p* < 0.001), and had higher illness severity scores for the APACHE (*p* = 0.001), SAPS (*p* < 0.001) and SOFA (*p* = 0.001) tests. They also had significantly higher levels of creatinine (*p* < 0.001) and lower rates of creatinine clearance (CCR (*p* < 0.001)) and GFR (*p* < 0.001). There was also a trend towards higher levels of IAP (*p* = 0.065) and urea (*p* = 0.075) in the group that died. Concerning BIA-derived raw data, non-survivors had significantly lower levels of reactance (*p* = 0.039) compared to those who survived. All other raw data were not found to be significantly different between survivors and non-survivors.

**Figure 5:**
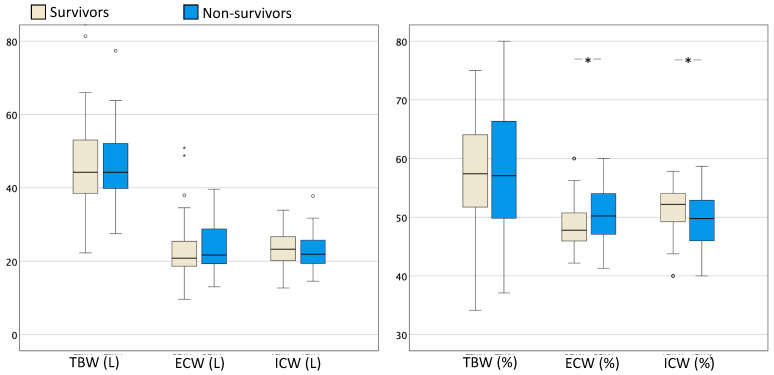
Box and whisker plots comparing body water composition with TBW, ECW, and ICW expressed in litres (**left panel**) and the percentage of TBW (**right panel**) in ICU survivors (*n* = 61) and non-survivors (*n* = 40). The error bars are the 95% confidence interval, the bottom and top of the box are the 25th and 75th percentiles, the line inside the box is the 50th percentile (median), and any outliers are shown as open circles. A *p* value < 0.05 between groups is indicated with *. ECW = extracellular water; ICW = intracellular water; TBW = total body water.

Additional variables were also assessed in both groups and subdivided into two categories: body fluid composition and nutritional status, as summarized in Table 5. Concerning body fluid composition, fluid overload (*p* = 0.033), ECW% (*p* = 0.047) and the ECW/ICW ratio (*p* = 0.049) were significantly higher in non-survivors. Accordingly, ICW% (*p* = 0.047) was significantly lower in non-survivors compared to survivors. Figure 5 illustrates the differences in body fluid composition in survivors vs. non-survivors. 

**Figure 6:**
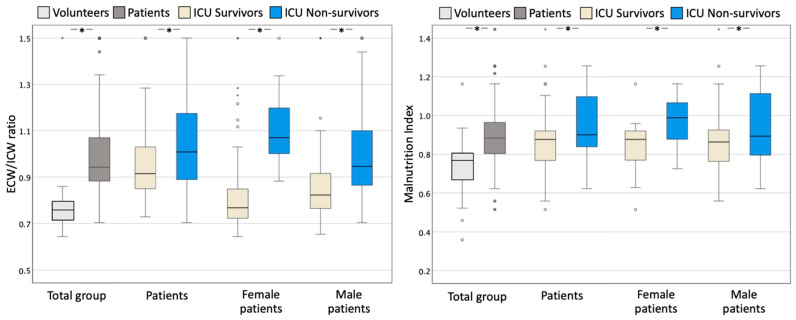
Box and whisker plots comparing the capillary leak index and malnutrition index with the ECW/ICW ratio (**left panel**) and the malnutrition index (**right panel**) in ICU survivors (*n* = 61) and non-survivors (*n* = 40). The error bars are the 95% confidence interval, the bottom and top of the box are the 25th and 75th percentiles, the line inside the box is the 50th percentile (median), and any outliers are shown as open circles. A *p* value < 0.05 between groups is indicated with *. ECW = extracellular water; ICW = intracellular water.

Concerning the nutritional status, the malnutrition index was significantly higher in non-survivors (*p* = 0.048), but all other variables were not found to be statistically different. Figure 6 illustrates the differences in the malnutrition index and the capillary leak index (defined by the ECW/ICW ratio) in healthy volunteers and patients, in ICU-survivors vs. non-survivors, and according to gender.

## 4. Discussion

This study has highlighted differences in BIA-derived measurements between healthy individuals and critically ill patients and between survivors and non-survivors in the ICU. 

### 4.1. Differences between Healthy Volunteers and ICU Patients

When comparing healthy individuals with ICU patients, we found a significantly higher impedance and lower Pha in ICU patients, which is consistent with previous findings [22]. Derivative parameters such as TBW, ECW, VE, and ECW/ICW ratio were all higher in ICU patients. This all points towards higher malnutrition and overhydration, with typical higher elevations in ECW in ICU patients [22,25]. This difference in volume state can be explained by the concept of capillary leak and hyperpermeability, which occurs in states of inflammation and results in the leakage of fluids from the intravascular to the extravascular compartment. Aggressive fluid management in critically ill patients can further exacerbate this fluid shift and contribute to fluid overload and accumulation, evidenced by an increase in TBW [26,27]. Clinical observation in 123 patients treated in two ICU centers documented a strong relationship between poor outcome and the fluid balance and capillary leak index, defined as the C-reactive protein (CRP) over albumin ratio [28]. Additionally, it has been documented that cumulative fluid balance (corresponding to fluid accumulation in the extravascular space, tissue edema, and subsequent elevated IAP) is significantly higher in non-survivors [28,29]. In this study we observed similar relationships, and capillary leak values measured as ECW/ICW and IAP were higher in non-survivors. Still, careful conclusions need to be made about BIA-derived findings, as some controversies exist [19,22,30]. More studies need to be performed in critically ill patients before a generalized use can be recommended [31].

### 4.2. Differences between Survivors and Non-Survivors

Differences in BIA-derived parameters between survivors and non-survivors in the ICU were analysed, and a statistically significant increase in ECW% and a concomitant decrease in ICW% in non-survivors was found. The ECW/ICW ratio was significantly higher in non-survivors, and there was a trend towards higher IAP values. These findings are consistent with the results of studies conducted by Yao et al. and Lee et al. [32,33].

Lee et al. reported that non-survivors had a higher ECW/TBW ratio, while Yao et al. found that survivors had a higher ratio ECW/weight ratio [32,33]. The differences in ECW and TBW between the two groups could be attributed to the fact that critically ill patients experience alterations in fluid composition in the different fluid compartments due to capillary leak and other pathological processes, which can lead to fluid accumulation. This fluid accumulation can, in turn, impact the patient’s prognosis (with venous congestion and abdominal hypertension), leading to end-organ dysfunction and failure and increased risk of mortality, which is then termed fluid accumulation syndrome [12]. We indeed found higher IAP values in non-survivors, albeit not statistically significant ones (*p* = 0.065); however, in patients who died in the hospital, IAP values were significantly (*p* = 0.05) increased (ESM Table S6). 

Another study by Lee et al., in critically ill patients also showed that several BIA-derived parameters, including ECW, TBW, ECW/TBW, and fat mass (FM), were related to mortality [34]. Similar results were obtained in other studies that linked changes in ECW/TBW to outcomes in diseases such as heart failure, renal disease, and liver disease [35,36]. Furthermore, it was reported that the ECW/TBW ratio was an independent factor associated with a prolonged duration of mechanical ventilation. This was confirmed by others, showing a better prognosis resulting from the management of edema and diminished ECW [20,37]. In contrast, Razerra et al., showed that ECW/TBW was not related to mortality in a group of eighteen ICU patients [38]. However, the authors noted that PhA, a parameter influenced by water distribution in cells and related to the ECW/ICW ratio, was associated with the mortality rate in numerous studies. Specifically, low PhA values have been associated with increased mortality in different conditions, including septic shock [25,39,40]. Diaz-De Los Santos et al. found that patients with septic shock and a PhA below six had an increased mortality rate [41]. Thibault et al. also demonstrated that an increase of one degree in PhA can result in dramatic changes in the mortality rate [42]. Our results confirm these findings as we found a significantly increased malnutrition index in non-survivors. A lower PhA was also found, but this was not statistically significant.

The relationship between lower ICW and mortality remains a controversial topic. While some studies [32,33], such as those conducted by Lee et al. and Yao et al., did not find an association between ICW and mortality, others, such as Vaara et al., found significant differences in TBW, ICW, and ECW between survivors and non-survivors in ICU [43].

To our knowledge, no other study has investigated the differences in BIA-derived parameters between healthy volunteers and critically ill patients, and further investigations are required to validate our findings.

It is possible that the conflicting results regarding ICW and mortality may be due to differences in study populations, sample sizes, and disease severity. Moreover, it is important to consider that BIA-derived parameters are influenced by a variety of factors, including age, gender, and body composition, among others. Therefore, it is necessary to take these factors into account when interpreting the results of BIA-derived parameters in critically ill patients.

We propose utilizing BIA as a simple and non-invasive approach to monitor parameters related to body and fluid composition during the management of fluid resuscitation. This method can serve as a valuable tool for both guidance and safety monitoring in terms of quality control and identification of potential side effects, but its limitations should be well known by the user, as will be discussed further.

### 4.3. Importance of Fluid Overload and Intra-Abdominal Hypertension

This study also revealed a statistically significant difference in fluid overload (FO) between survivors and non-survivors, which may be explained by endothelial dysfunction and capillary leak commonly observed in patients with sepsis [7]. This can result in the accumulation of excess fluid in the abdominal cavity, leading to organ compression, increased intra-abdominal pressure (IAP), and ultimately increased morbidity and mortality [10]. Direct measurement of IAP can be achieved through a needle or catheter inserted in the peritoneal space, while indirect measurement can be accomplished through an indwelling bladder catheter [44]. Since FO is correlated with IAP and is associated with poor outcomes, BIA may be a potential non-invasive tool for monitoring critically ill patients, leading to interventions to reduce the cumulative fluid balance in these patients [45,46]. However, it is important to remain cautious when interpreting the results; despite significant differences in BIA-derived parameters between survivors and non-survivors in the ICU being observed in this study, a logistic regression analysis previously revealed that the SAPS score was the sole significant predictor of mortality in the ICU [7]. This score is an estimation of the severity of the disease that requires ICU admission based on different variables such as age, physiological measures, need for ventilation, consciousness, and laboratory results. The SAPS-II score has been widely used in the ICU setting and has been shown to be a useful tool for predicting mortality and assessing the severity of illness [47]. 

Several studies have documented that fluid overload and accumulation can affect BIA-derived variables that correspond to tissue edema and IAH [21,48,49]. An examination of the fluid and body balance in children undergoing hemodialysis revealed a significant association between FO and VE. Furthermore, an increase in VE was found to be linked with various clinical manifestations, including pitting edema and hypertension [49]. A separate clinical investigation reported a robust correlation between heightened IAP and excess ECW, and the latter significantly increases the likelihood of IAH. The study revealed that ECW levels exceeding 22.4 L in critically ill patients and 24.9 L in surgical patients pose a high risk for IAH [48]. Notably, an increase in ECW affects TBW, which may result from the traumatic insult of surgery in combination with aggressive fluid therapy and increased capillary permeability in patients undergoing open major abdominal surgery [21]. A similar relationship was found in critically ill patients with liver cirrhosis, heart failure, and kidney failure [30].

Recent (systematic) reviews have pointed towards the issues in the validation of BIA-derived parameters against the gold standard, as the calculation of fluid composition and cumulative fluid balance is not without error or bias in critically ill patients [19,22,30]. Therefore it was not surprising that bioelectrical impedance analysis had no systematic errors or bias, but wide limits of agreement [50]. On the other hand changes in bioimpedance were correlated with the duration of overall organ failure, circulatory failure, and fluid status. Single measurements of bioimpedance were not associated with any changes in organ dysfunction [51]. For a new device to be validated as an evidence-based monitor, four basic questions need to be answered: first, does the device perform as well as the gold standard; second, does the device provide the clinician with new (calculated or measured) data or information; third, does this new information alter our treatment at the bedside; and finally, will this change in treatment eventually affect outcomes positively. The present study (with all its limitations) concerns the second question, and it seems that the phase angle, malnutrition index, and ECW to ICW ratio provide promising additional information. However further prospective validation in critically ill patients is needed.

### 4.4. Study Limitations

This study has several limitations. Firstly, the study is limited by a relatively small sample size and its retrospective design. It is important to recognize that the associations observed in this study do not establish causation. The retrospective nature of the study means that it relies on existing data from BIA measurements performed at the discretion of the attending physician and does not involve any manipulation or control of variables. Therefore, we cannot rule out the possibility that other factors may have influenced the observed associations. Moreover, a larger sample size would have provided more statistical power to identify potential causative factors.

Secondly, there are limitations in how the body composition parameters were obtained. For example, parameters such as height and body weight are needed to calculate data such as ECW, ICW, and TBW. The accuracy of these measurements needs to be validated prior to using them, as there are different reasons for inaccurate measurements, such as the use of multiple devices attached to a patient in ICU that can provide falsified results for weight [33]. Moreover, differences in hydration status can influence the calculations of TBW, and the equations for TBW have been developed for people with normal hydration status, which may not be applicable in critically ill patients with altered hydration [52]. Therefore, clinicians need to be cautious when interpreting results related to TBW.

Thirdly, we need to acknowledge that our BIA measurements were performed only once, on average on Day 5 within the first week of the ICU stay. This may have caused a selection bias, as patients who died earlier or were discharged earlier from ICU may have been missed. Moreover, changes in body composition parameters over time were not monitored, and we cannot exclude the possibility of type I or type II errors in our findings.

Fourthly, patient selection and demographics were not fully described in our study, as we could not always collect full information on the admission diagnoses. However, we calculated severity scores and found significant differences between survivors and non-survivors, which may suggest that disease severity played a role in the differences observed in body composition parameters. Future studies should include more comprehensive information on patient demographics and admission diagnoses to better understand the associations between body composition and clinical outcomes in critically ill patients.

A final limitation of this study is the observed difference in patient and healthy subject demographics. The healthy subgroup had more females, was younger, and showed a higher BMI, which could affect some of the BIA-derived parameters. Therefore, the readers should consider these differences when analyzing and interpreting the results, and future studies should aim to have a more balanced representation of both genders and age groups.

Overall, these limitations suggest the need for caution when interpreting the results of this study. Further research is necessary to validate the accuracy of the measurements used in this study and to investigate the impact of gender and age differences on BIA-derived parameters in critically ill patients. Furthermore, this study opens new avenues for future research, including investigating the relationship between BIA-derived parameters and clinical outcomes, such as length of stay in the ICU and mechanical ventilation duration.

## 5. Conclusions

BIA is an easy, fast, and cost-effective tool to investigate body fluid composition in critically ill patients but should be used with care, as a large number of confounding factors and variables make its prognostic value uncertain. When comparing ICU patients with healthy subjects, this study observed that ICU patients have a higher TBW with an accumulation in the extracellular compartment. ICU non-survivors showed similar results, indicating that ICU patients and a fortiori non-survivors are generally overhydrated, with accumulation of ECW, and more undernourished, as indicated by a higher malnutrition index.

## 6. Take Home Messages

- Overall, this study contributes to the growing body of literature on the utility of BIA-derived parameters in critically ill patients and underscores the impact of inflammation and capillary leak on fluid distribution in this population.
- This study highlights the importance of considering gender-specific differences in BIA-derived parameters when assessing fluid status in healthy volunteers as well as critically ill patients. By considering these differences, clinicians can tailor their fluid management strategies more effectively to optimize patient outcomes.
- The results add to the growing body of evidence that BIA-derived parameters (showing a decrease in ICW and increase in ECW and the ECW/ICW ratio) can provide valuable insights into the fluid status of critically ill patients.
- We propose to identify capillary leak as an increased ECW/ICW ratio above 0.9, as was shown with the AUROC analysis.
- Findings highlight the importance of using a comprehensive approach that considers clinical factors, such as the SAPS-II score, when assessing the prognosis of ICU patients.
- Findings also highlight the importance of PhA and the malnutrittion index as prognostic factors in critically ill patients and suggest that they may be a useful addition to routine BIA-derived parameters for assessing mortality risk in ICU patients.
- We identified a malnutrition index above 0.8 to be associated with poor outcomes.
- While the relationship between ICW and mortality remains unclear, this study demonstrated that several BIA-derived parameters, including ECW/ICW, were related to mortality in ICU patients.
- Further studies are needed to validate these findings and to better understand the relationship between BIA-derived parameters and clinical outcomes in critically ill patients.

## Figures and Tables

**Table 1:** Demographic data and raw BIA values at 50 kHz. Means and standard deviations (SD) are listed for both female (*n* = 35) and male (*n* = 66) ICU patients (*n* = 101) and female (*n* = 68) and male (*n* = 33) healthy volunteers (*n* = 101).

	Female			Male		
Variable	Healthy Volunteers (*n* = 68)	ICU Patients (*n* = 35)	*p*-Value	Healthy Volunteers (*n* = 33)	ICU Patients (*n* = 66)	*p*-Value
	Patient demographics					
Height (cm)	169.3 ± 8.1	163.7 ± 5.5	<0.001	181.0 ± 6.6	175.5 ± 8.3	<0.001
Weight (kg)	67.4 ± 12.4	77.4 ± 20.4	0.005	83.1 ± 14.3	84.0 ± 20.4	NS
BMI (kg m^−2^)	23.4 ± 3.3	28.9 ± 7.3	<0.001	25.3 ± 4.0	27.3 ± 6.7	NS
Age (years)	37.6 ± 12.6	60.3 ± 16.9	<0.001	38.9 ± 12.5	65.2 ± 14.8	<0.001
	Raw BIA values at 50 kHz					
Impedance (Ohm)	586.8 ± 105.3	404.7 ± 113.7	<0.001	506.9 ± 62.6	381.7 ± 96.4	<0.001
Phase angle	9.3 ± 1.5	7.9 ± 1.5	<0.001	9.5 ± 1.0	8.6 ± 3.8	0.038
Resistance (Ohm)	579.5 ± 104.2	401.3 ± 112.8	<0.001	500.5 ± 61.6	377.3 ± 96.1	<0.001
Reactance (Ohm)	94.2 ± 20.6	55.1 ± 17.9	<0.001	83.6 ± 14.2	56.4 ± 24.7	<0.001
Capacitance (Ohm)	35.7 ± 11.3	63.6 ± 20.4	<0.001	39.0 + 5.0	65.4 ± 25.2	<0.001

The *p*-value indicates the statistical significance of the differences observed between the two groups. BMI—Body mass index.

**Table 2:** Results of body fluid composition for female and male ICU patients (*n* = 101) and healthy volunteers (*n* = 101).

Female				Male		
Variable	Healthy Volunteers (*n* = 68)	ICU Patients (*n* = 35)	*p*-Value	Healthy Volunteers (*n* = 33)	ICU Patients (*n* = 66)	*p*-Value
	Body fluid composition					
Dry Weight (kg)	67.8 ± 13.4	72.4 ± 18.7	NS	83.1 ± 14.3	78.1 ± 18.9	NS
Volume excess (L)	−0.2 ± 0.7	5.0 ± 5.2	<0.001	−0.0 ± 1.0	5.9 ± 6.6	<0.001
Fluid Overload (%)	0.0 ± 0.1	6.2 ± 5.8	<0.001	0.0 ± 0.1	6.9 ± 7.0	<0.001
TBW (L)	35.0 ± 6.7	40.2 ± 8.6	0.003	45.7 ± 5.0	49.6 ± 10.3	0.013
TBW (%)	52.0 ± 5.5	53.1 ± 9.2	NS	55.7 ± 5.7	60.2 ± 9.3	0.002
ECW (L)	15.2 ± 3.7	20.5 ± 5.7	<0.001	19.8 ± 2.5	24.6 ±7.0	<0.001
ECW (%)	43.2 ± 2.7	50.6 ± 4.3	<0.001	43.2 ± 1.7	49.1 ± 4.8	<0.001
ICW (L)	19.8 ± 3.4	19.7 ± 3.6	NS	25.9 ± 2.8	25.0 ± 4.2	NS
ICW (%)	56.8 ± 2.7	49.4 ± 4.3	<0.001	56.8 ± 1.7	50.9 ± 4.8	<0.001
ECW/ICW	0.8 ± 0.1	1.0 ± 0.2	<0.001	0.8 ± 0.1	1.0 ± 0.2	<0.001
ECS (L)	5.2 ± 0.9	4.8 ± 0.8	NS	6.8 ± 0.7	6.3 ± 1.0	0,006
ECF (L)	15.8 ± 3.8	21.4 ± 5.9	<0.001	20.6 ± 2.6	25.6 ± 7.3	<0.001
Plasma Fluid (L)	3.0 ± 0.8	4.0 ± 1.2	<0.001	4.0 ± 0.6	4.7 ± 1.4	0.001
Interstitial Fluid (L)	11.3 ± 2.7	15.3 ± 4.2	<0.001	14.5 ± 1.8	18.5 ± 5.3	<0.001
FFMH (%)	72.7 ± 2.4	79.0 ± 4.3	<0.001	72.5 ± 2.1	78.1 ± 4.3	<0.001
Body Density (Kg/L)	1.04 ± 0.01	1.03 ± 0.02	0.026	1.05 ± 0.02	1.05 ± 0.02	NS

The *p*-value indicates the statistical significance of the differences observed between the two groups. ECF—extracellular fluid, ECS—extracellular solids, ECW—extracellular water, FFMH—fat-free mass hydration, ICW—intracellular water, TBW—total body water.

**Table 3:** Results of the nutritional status assessment for female and male ICU patients (*n* = 101) and healthy volunteers (*n* = 101).

Female				Male		
Variable	Healthy Volunteers (*n* = 68)	ICU Patients (*n* = 35)	*p*-Value	Healthy Volunteers (*n* = 33)	ICU Patients (*n* = 66)	*p*-Value
	Nutritional Status					
Phase angle	9.3 ± 1.5	7.9 ± 1.5	<0.001	9.5 ± 1.0	8.6 ± 3.8	0.038
Malnutrition Index	0.73 ± 0.12	0.89 ± 0.14	<0.001	0.77 ± 0.08	0.90 ± 0.18	<0.001
RMR (kcal)	1571.2 ± 205.9	1445.8 ± 186.4	0.003	1971.5 ± 202.2	1753.0 ± 282.4	<0.001
FFM (kg)	48.1 ± 8.5	50.6 ± 9.0	NS	63.0 ± 6.3	63.4 ± 11.3	NS
FFM (%)	71.5 ± 6.5	67.1 ± 10.2	0.025	76.8 ± 7.4	76.8 ± 8.9	NS
Fat (kg)	19.8 ± 7.4	26.8 ± 15.4	0.014	20.1 ± 10.5	20.6 ± 12.2	NS
Fat (%)	28.5 ± 6.5	32.9 ± 10.2	0.025	23.2 ± 7.4	23.1 ± 8.9	NS
BCM (kg)	28.0 ± 5.1	26.9 ± 5.5	NS	35.6 ± 4.0	33.5 ± 6.0	0.035
ECM (kg)	20.1 ± 4.4	23.7 ± 4.7	<0.001	27.4 ± 3.1	29.9 ± 6.9	0.014
Protein (kg)	11.2 ± 2.0	10.9 ± 2.1	NS	14.4 ± 1.5	13.4 ± 2.4	0.013
Mineral (kg)	4.4 ± 0.6	4.3 ± 0.9	NS	5.0 ± 0.5	4.7 ± 0.8	0.014
Muscle (kg)	24.6 ± 5.2	22.8 ± 5.1	NS	33.0 ± 3.5	30.0 ± 5.5	0.001
TBK (g)	124.1 ± 25.0	116.2 ± 22.9	NS	169.1 ± 17.7	154.7 ± 26.7	0.006
TBCa (g)	1020.5 ± 180.6	963.6 ± 165.4	NS	1346.0 ± 128.2	1241.9 ± 192.9	0.006
Glycogen (g)	468.5 ± 82.3	442.7 ± 87.2	NS	599.8 ± 63.0	545.1 ± 94.1	0.003

The *p*-value indicates the statistical significance of the differences observed between the two groups. BCM—body cell mass, ECM—extracellular mass, FFM—fat-free mass, RMR—resting metabolic rate, TBCa—total body calcium, TBK—total body kalium.

**Table 4:** Demographic data, laboratory data, and raw BIA values for survivors (*n* = 61) and non-survivors (*n* = 40) in the ICU.

Variable	Total	Alive (*n* = 61)	Died (*n* = 40)	*p*-Value
Patient demographics				
Male/female	2/1	2/1	2/1	NS
Hospital stay (days)	51.9 ± 47.5	65.5 ± 53.3	31 ± 26.2	<0.001
ICU stay (days)	31.2 ± 26.7	34.6 ± 28.8	26 ± 22.4	NS
Day measurement	4.8 ± 2.1	5 ± 2.1	4.6 ± 2.2	NS
Height (cm)	171.4 ± 9.4	172.1 ± 9.3	170.4 ± 9.5	NS
Weight (kg)	81.7 ± 20.6	82.9 ± 21.9	79.9 ± 18.6	NS
BMI (kg/m^2^)	27.8 ± 6.9	28 ± 7.1	27.6 ± 6.7	NS
Age (years)	63.5 ± 15.7	60.1 ± 16.6	68.7 ± 12.7	0.007
APACHE	23.3 ± 9.1	21 ± 8.7	26.8 ± 8.6	0.001
SAPS	55.5 ± 18.9	49.1 ± 16.8	65 ± 17.9	<0.001
SOFA	9.8 ± 4.1	8.7 ± 3.2	11.4 ± 4.7	0.001
IAP (mmHg)	13.2 ± 3.9	12.5 ± 3.8	14.1 ± 4	0.065
EVLWI (mL/kgPBW)	10.6 ± 3.2	10.4 ± 2.7	10.8 ± 3.8	NS
Laboratory results				
Hematocrit (%)	28.9 ± 5.8	29.2 ± 5.8	28.4 ± 5.9	NS
Total protein (g/L)	49.6 ± 8.4	48.7 ± 8.6	51 ± 8.1	NS
Albumin (g/L)	24.5 ± 5.2	24.7 ± 6.2	24.2 ± 3.5	NS
CRP (mg/dL)	164.2 ± 113.6	169.4 ± 114.4	156.5 ± 113.5	NS
Urea (mg/DL)	69.6 ± 45.9	62.8 ± 43.8	79.5 ± 47.5	0.075
Osmol (measured)	298 ± 18	297.3 ± 19.8	299.3 ± 14.7	NS
Osmol (calculated)	314.4 ± 40.4	309.2 ± 48	322.1 ± 23.4	NS
Glucose (mg/dL)	142.8 ± 52.2	142.4 ± 52.2	143.4 ± 52.9	NS
Na (mmol/L)	142.4 ± 8	142.1 ± 8.8	142.9 ± 6.7	NS
K (mmol/L)	4.2 ± 0.6	4.2 ± 0.6	4.1 ± 0.6	NS
Creatine (mg/dL)	1.3 ± 1	1.1 ± 0.6	1.8 ± 1.2	<0.001
CCR (mL/min)	99.5 ± 48.8	113.4 ± 48.3	78.3 ± 41.7	<0.001
GFR (mL/min)	82.6 ± 45.4	95.1 ± 44.8	63.6 ± 39.7	<0.001
Raw BIA values at 50 kHz				
Impedance (Ohm)	389.7 ± 102.8	401 ± 99	372.4 ± 107.1	NS
Phase angle	8.3 ± 3.2	8.7 ± 3.8	7.8 ± 2.2	NS
Resistance (Ohm)	385.6 ± 102.2	396.4 ± 99.1	369.3 ± 106.1	NS
Reactance (Ohm)	55.9 ± 22.5	59.7 ± 23.8	50.2 ± 19.4	0.039
Capacitance (Ohm)	64.8 ± 23.5	60 ± 20.9	72.1 ± 25.6	0.010

The *p*-value indicates the statistical significance of the differences observed between the two groups. APACHE—acute physiology and chronic health evaluation; BMI—body mass index; CCR—creatinine clearance rate; CRP—C-reactive protein; EVLWI—extravascular lung water index; GFR—glomerular filtration rate; IAP—intra-abdominal pressure; ICU—intensive care unit; SOFA—sequential and organ failure assessment.

**Table 5:** Body fluid composition and nutritional status between survivors (*n* = 61) and non-survivors (*n* = 40) in the ICU.

Variable	Total	Alive (*n* = 61)	Died (*n* = 40)	*p*-Value
Body fluid composition				
Dry Weight (kg)	76.1 ± 19	77.9 ± 18.9	73.4 ± 19	NS
Cumulative FB (L)	7.1 ± 6.2	7 ± 6.1	7.2 ± 6.5	NS
Volume excess (L)	5.6 ± 6.1	5 ± 6.4	6.5 ± 5.7	NS
Fluid Overload (%)	6.7 ± 6.6	5.5 ± 5.5	8.4 ± 7.6	0.033
TBW (L)	46.3 ± 10.7	46.6 ± 11.3	46 ± 9.8	NS
TBW (%)	57.8 ± 9.8	57.2 ± 9.5	58.6 ± 10.3	NS
ECW (L)	23.2 ± 6.8	23 ± 7.3	23.5 ± 6.1	NS
ECW (%)	49.6 ± 4.7	48.9 ± 4.3	50.7 ± 5.1	0.047
ICW (L)	23.1 ± 4.8	23.6 ± 4.8	22.5 ± 4.7	NS
ICW (%)	50.4 ± 4.7	51.1 ± 4.3	49.2 ± 5.1	0.047
ECW/ICW	1 ± 0.2	0.97 ± 0.19	1.05 ± 0.22	0.049
ECS (L)	5.8 ± 1.2	5.9 ± 1.2	5.6 ± 1.1	NS
ECF (L)	24.1 ± 7.1	23.9 ± 7.6	24.4 ± 6.3	NS
Plasma Fluid (L)	4.5 ± 1.4	4.5 ± 1.5	4.5 ± 1.1	NS
Interstitial Fluid (L)	17.4 ± 5.2	17.1 ± 5.4	17.7 ± 4.9	NS
FFMH (%)	78.4 ± 4.3	78 ± 4	79 ± 4.7	NS
Body Density (kg/L)	1.039 ± 0.023	1.039 ± 0.023	1.04 ± 0.022	NS
Nutritional status				
Phase angle	8.3 ± 3.2	8.7 ± 3.8	7.8 ± 2.2	NS
Malnutrition Index	0.9 ± 0.16	0.87 ± 0.16	0.94 ± 0.17	0.048
RMR (kcal)	1646.5 ± 291.9	1680.1 ± 306.5	1595.3 ± 263.6	NS
FFM (kg)	58.9 ± 12.2	59.5 ± 12.7	58.1 ± 11.3	NS
FFM (%)	73.5 ± 10.4	73.2 ± 10.6	73.9 ± 10.2	NS
Fat (kg)	22.8 ± 13.7	23.4 ± 14.6	21.8 ± 12.1	NS
Fat (%)	26.5 ± 10.4	26.8 ± 10.6	26.2 ± 10.2	NS
BCM (kg)	31.2 ± 6.6	31.8 ± 6.4	30.2 ± 6.9	NS
ECM (kg)	27.7 ± 6.8	27.7 ± 7.5	27.9 ± 5.8	NS
Protein (kg)	12.5 ± 2.6	12.8 ± 2.6	12.1 ± 2.6	NS
Mineral (kg)	4.6 ± 0.9	4.7 ± 0.9	4.4 ± 0.8	NS
Muscle (kg)	27.5 ± 6.3	28.1 ± 6.3	26.5 ± 6.2	NS
TBK (g)	141.4 ± 31.3	145 ± 32.1	135.9 ± 29.5	NS
TBCa (g)	1145.4 ± 226.3	1171.4 ± 232.4	1105.9 ± 213.4	NS
Glycogen (g)	509.6 ± 103.6	523.6 ± 106.4	488.2 ± 96.7	0.092

The *p*-value indicates the statistical significance of the differences observed between the two groups. BCM—body cell mass; ECF—extracellular fluid; ECM—extracellular mass; ECS—extracellular solids; ECW—extracellular water; FAT%—fat percentage; FFM—fat-free mass; FFMH—fat-free mass hydration; ICW—intracellular water; RMR—resting metabolic rate; TBCa—total body calcium; TBK—total body kalium; TBW = total body water.

## Data Availability

The data presented in this study are not publicly available but are available on request from the corresponding author.

## Supplementary Materials

The following supporting information can be downloaded at: https://www.mdpi.com/article/10.3390/life14010027/s1, ESM Table S1: Comparison of demographic data between ICU patients vs. healthy volunteers; ESM Table S2: Comparison of raw data between ICU patients vs. healthy volunteers; ESM Table S3: Comparison of body fluid composition between ICU patients vs. healthy volunteers; ESM Table S4: Comparison of nutritional status between ICU patients vs. healthy volunteers; ESM Table S5: Comparison of demographic and bioelectrical impedance analysis (BIA) variables between female and male volunteers and patients; ESM Table S6: Demographics, laboratory results, and raw BIA parameters in hospital survivors vs. non-survivors; ESM Table S7: Body fluid composition and nutritional status in hospital survivors vs. non-survivors; ESM Figure S1: Boxplots comparing body water distribution in female and male patients. Refs. [53,54] are cited in the Supplementary Materials.

## Abbreviations

APACHE II	Acute Physiology and Chronic Health Evaluation II
BIA	Bio-electrical impedance analysis
BIVA	Bioelectrical impedance vector analysis
BVA	Blood volume assessment
BCM	Body cell mass
BMI	Body mass index
CCR	Creatinine clearance rate
COPD	Chronic obstructive pulmonary disease
CRP	C-reactive protein
DEXA	Dual-energy X-ray absorptiometry
ECF	Extracellular fluid
ECS	Extracellular solids
ECW	Extracellular water
ECW%	Extracellular water percentage
FA	Fluid accumulation
FAS	Fluid accumulation syndrome
FAT%	Fat in percentage
FFM	Fat-free mass
FFMH%	Fat-free mass hydration percentage
GFR	Glomerular filtration rate
FO	Fluid overload
Hct	Haematocrit
IAP	Intra-abdominal pressure
IAH	Intra-abdominal hypertension
ICU	Intensive care unit
ICW	Intracellular water
ICW%	Intracellular water percentage
PhA	Phase angle
RMR	Resting metabolic rate
ROC	Receiver operating characteristics
SAPS	Simplified Acute Physiology Score
SOFA	Sequential Organ Failure Assessment
TBCa	Total body calcium
TBK	Total body potassium
TBW	Total body water
VE	Volume excess
ZNA	Ziekenhuisnetwerk Antwerpen
