# Supplementary material for: Comparison of Bioelectrical Impedance Analysis (BIA)-Derived Parameters in Healthy Volunteers and Critically Ill Patients
Source: Life (Basel). 2023 Dec 23;14(1):27. doi: 10.3390/life14010027 (PMC10821189; doi:10.3390/life14010027)
Supplement: Supplementary file 1 [file life-14-00027-s001.zip › life-2354255-supplementary.pdf]

life

MDPI

Electronic Supplemental Material (ESM)

1. Comparison Between ICU Patients and Healthy Volunteers

In total, 235 BIA measurements were obtained in 101 healthy individuals and 101 patients. Of the 101 patients, 65% were male, whereas in the healthy group only 35% were male. ESM Table S1 represents the demographic data as well as the raw BIA values at 50 kHz.

ESM Table S1. Comparison of demographic data between ICU patients and healthy volunteers. Demographic data and raw BIA values. Means and SD are listed for both ICU patients (n=101) and healthy volunteers (n=101). The p-value indicates the statistical significance of the differences observed between the two groups. *BMI – body mass index*

Variable	Total	Heathy volunteers (n=101)	ICU patients (n=101)	*p*-value
		**Patient demographics**		
Male/Female	1/1	1/2	2/1	0.010
Height (cm)	172.3 ± 9.4	173.1 ± 9.4	171.4 ± 9.4	NS
Weight (kg)	77.1 ± 18.5	72.5 ± 15	81.7 ± 20.6	0.000
BMI (kg m^{-2})	25.9 ± 5.8	24 ± 3.6	27.8 ± 6.9	0.000
Age (years)	50.8 ± 19	38 ± 12.5	63.5 ± 15.7	0.000
		**Raw BIA values at 50 kHz**		
Impedance (Ohm)	475.2 ± 132.8	560.7 ± 100.5	389.7 ± 102.8	0.000
Phase angle	8.8 ± 2.5	9.3 ± 1.3	8.3 ± 3.2	0.005
Resistance (Ohm)	469.7 ± 131.2	553.7 ± 99.4	385.6 ± 102.2	0.000
Reactance (Ohm)	73.3 ± 27.3	90.8 ± 19.3	55.9 ± 22.5	0.000
Capacitance (Ohm)	50.8 ± 22.9	36.8 ± 10	64.8 ± 23.5	0.000

In terms of patient demographics, the male/female ratio was significantly different between the healthy volunteers (1/2) and ICU patients (2/1) (p=0.010). The ICU patients were also significantly older than the healthy volunteers (p=0.000). There was no significant difference in height between the two groups, but the ICU patients had a significantly higher weight and BMI compared to the healthy volunteers (p=0.000 for both).

Regarding BIA values, the ICU-patients had significantly lower impedance, resistance, and reactance but a higher capacitance at 50 kHz compared to healthy individuals (p=0.000 for all). The phase angle was also significantly lower in ICU patients compared to healthy volunteers (p=0.005). Significant differences in the raw data of impedance, capacitance, reactance, and resistance were observed at each of the four frequencies (5, 50, 100 and 200 kHz) (see also electronic supplemental material, ESM Table S2). ESM Table S1 lists the results obtained at the 50 kHz frequency.

ESM Table S3 presents the results of the body fluid composition, showing that ICU patients had significantly higher VE, FO, TBW, ECW, ICW, ECW/ICW ratio, extracellular fluid (ECF), interstitial fluid, and FFMH% values compared to healthy individuals (p=0.000 for all). On the other hand, the healthy individuals had a significantly higher ICW (L and %) compared to the ICU patients (p=0.037 and p=0.000, respectively). There was no significant difference in dry weight, body density, or extracellular solids (ECS) between the two groups.

ESM Table S2: Comparison of raw data between ICU patients and healthy volunteers. Raw BIA data analysis at multiple frequencies.

Variable	Total	Heathy volunteers (n=101)	ICU patients (n=101)	*p*-value
**Raw BIA values at 50 kHz**				
Impedance (Ohm)	547.8 ± 164.3	655.5 ± 117.3	440 ± 130.5	0.000
Phase angle	3.3 ± 4	3.3 ± 2	3.4 ± 5.4	NS
Resistance (Ohm)	546.1 ± 164.6	654.9 ± 117.9	437.4 ± 129.2	0.000
Reactance (Ohm)	31.5 ± 34.8	35.8 ± 14.3	27.1 ± 46.8	0.075
Capacitance (Ohm)	1766.2 ± 1568.5	962.1 ± 281.4	2570.3 ± 1886.7	0.000
**Raw BIA values at 50 kHz**				
Impedance (Ohm)	475.2 ± 132.8	560.7 ± 100.5	389.7 ± 102.8	0.000
Phase angle	8.8 ± 2.5	9.3 ± 1.3	8.3 ± 3.2	0.005
Resistance (Ohm)	469.7 ± 131.2	553.7 ± 99.4	385.6 ± 102.2	0.000
Reactance (Ohm)	73.3 ± 27.3	90.8 ± 19.3	55.9 ± 22.5	0.000
Capacitance (Ohm)	50.8 ± 22.9	36.8 ± 10	64.8 ± 23.5	0.000
**Raw BIA values at 50 kHz**				
Impedance (Ohm)	444 ± 125	522.6 ± 96.8	365.4 ± 97.7	0.000
Phase angle	11.1 ± 2.6	11.2 ± 1.9	11.1 ± 3.1	NS
Resistance (Ohm)	435.7 ± 122.8	512.8 ± 95	358.7 ± 96.3	0.000
Reactance (Ohm)	85.6 ± 29.6	101.4 ± 25.8	69.9 ± 24.6	0.000
Capacitance (Ohm)	21.1 ± 8.7	16.7 ± 5.3	25.6 ± 9.3	0.000
**Raw BIA values at 50 kHz**				
Impedance (Ohm)	416.6 ± 115.7	490.6 ± 86	342.5 ± 91.9	0.000
Phase angle	14.2 ± 3	13.7 ± 2.8	14.7 ± 3.1	0.020
Resistance (Ohm)	403.9 ± 112.5	476.4 ± 83.2	331.3 ± 88.9	0.000
Reactance (Ohm)	102 ± 34.3	116.9 ± 32.3	87.1 ± 29.6	0.000
Capacitance (Ohm)	8.8 ± 3.6	7.3 ± 2.3	10.3 ± 4	NS

ESM Table S3. Comparison of body fluid composition between ICU patients and healthy volunteers. Results of the body fluid composition between a group of healthy volunteers (n=101)

Variable	Total	Heathy volunteers (n=101)	ICU patients (n=101)	*p*-value
**Body fluid composition**				
Dry Weight (kg)	74.5 ± 17.3	72.8 ± 15.4	76.1 ± 19	NS
Volume excess (L)	2.7 ± 5.2	-0.2 ± 0.8	5.6 ± 6.1	0.000
Fluid Overload (%)	3.2 ± 5.8	-0.3 ± 1.2	6.7 ± 6.6	0.000
TBW (L)	42.4 ± 10.2	38.5 ± 8	46.3 ± 10.7	0.000
TBW (%)	55.5 ± 8.4	53.2 ± 5.8	57.8 ± 9.8	0.000
ECW (L)	19.9 ± 6.5	16.7 ± 4	23.2 ± 6.8	0.000
ECW (%)	46.4 ± 4.9	43.2 ± 2.4	49.6 ± 4.7	0.000
ICW (L)	22.5 ± 4.6	21.8 ± 4.3	23.1 ± 4.8	0.037
ICW (%)	53.6 ± 4.9	56.8 ± 2.4	50.4 ± 4.7	0.000
ECW/ICW	0.9 ± 0.2	0.8 ± 0.1	1 ± 0.2	0.000
ECS (L)	5.8 ± 1.1	5.7 ± 1.1	5.8 ± 1.2	NS
ECF (L)	20.7 ± 6.7	17.4 ± 4.1	24.1 ± 7.1	0.000
Plasma Fluid (L)	3.9 ± 1.3	3.3 ± 0.9	4.5 ± 1.4	0.000
Interstitial Fluid (L)	14.9 ± 4.9	12.3 ± 2.9	17.4 ± 5.2	0.000
FFMH (%)	75.5 ± 4.5	72.6 ± 2.3	78.4 ± 4.3	0.000
Body Density (Kg/L)	1.039 ± 0.019	1.038 ± 0.016	1.039 ± 0.023	NS

and a group of ICU patients (n=101). The p-value indicates the statistical significance of the differences observed between the two groups. *ECF – extracellular fluid, ECS – extracellular solids, ECW –*

extracellular water, FFMH – fat-free mass hydration, ICW – intracellular water, TBW – total body water, VE – volume excess.

ESM Table S4 shows the comparison of nutritional status between healthy volunteers and ICU patients. The variables measured include the phase angle, malnutrition index, resting metabolic rate (RMR), fat-free mass (FFM), body cell mass (BCM), extracellular mass (ECM), fat mass, protein, mineral, muscle, total body potassium (TBK), total body calcium (TBCa), and glycogen values. Results indicate that ICU patients have a significantly lower phase angle and a higher malnutrition index compared to healthy volunteers. FFM and ECM are significantly lower in ICU patients, while fat mass is higher, although not statistically significant. All other findings were not found to be statistically different between the two groups.

ESM Table S4. Comparison of nutritional status between ICU patients and healthy volunteers. Results of the nutritional status assessment between a group of healthy volunteers (n=101) and a group of ICU patients (n=101). The p-value indicates the statistical significance of the differences

Variable	Total	Heathy volunteers (n=101)	ICU patients (n=101)	*p* -value
		**Nutritional status**		
Phase angle	8.8 ± 2.5	9.3 ± 1.3	8.3 ± 3.2	0.005
Malnutrition Index	0.8 ± 0.2	0.7 ± 0.1	0.9 ± 0.2	0.000
RMR (kcal)	1674.3 ± 285.5	1702 ± 277.7	1646.5 ± 291.9	NS
FFM (kg)	55.9 ± 11.7	53 ± 10.5	58.9 ± 12.2	0.000
FFM (%)	73.4 ± 8.9	73.2 ± 7.2	73.5 ± 10.4	NS
Fat (kg)	21.3 ± 11.4	19.9 ± 8.5	22.8 ± 13.7	0.072
Fat (%)	26.7 ± 8.9	26.8 ± 7.2	26.5 ± 10.4	NS
BCM (kg)	30.8 ± 6.3	30.5 ± 5.9	31.2 ± 6.6	NS
ECM (kg)	25.1 ± 6.7	22.5 ± 5.3	27.7 ± 6.8	0.000
Protein (kg)	12.4 ± 2.5	12.2 ± 2.4	12.5 ± 2.6	NS
Mineral (kg)	4.6 ± 0.8	4.6 ± 0.7	4.6 ± 0.9	NS
Muscle (kg)	27.4 ± 6.2	27.3 ± 6.1	27.5 ± 6.3	NS
TBK (g)	140.1 ± 31.2	138.8 ± 31.1	141.4 ± 31.3	NS
TBCa (g)	1136.1 ± 225.3	1126.9 ± 225	1145.4 ± 226.3	NS
Glycogen (g)	510.5 ± 100.7	511.4 ± 98.2	509.6 ± 103.6	NS

observed between the two groups. *BCM – body cell mass, ECM – extracellular mass, FFM – fat-free mass, RMR – resting metabolic rate, TBCa – total body calcium, TBK – total body kalium*

2. Female vs. Male Volunteers and Patients

Of the 101 volunteers, 68 were female and 35 were male, while of the 101 patients, 36 were female and 65 were male. This resulted in a significant ($p<0.01$) difference in the male-to-female ratio in volunteers compared to patients (ESM Table S1). ESM Table S5 presents the demographic details and BIA variables between the groups.

In terms of overall demographics, female volunteers were significantly shorter, lighter, and had lower BMIs than male volunteers ($p<0.05$). However, there was no significant difference in age between the two groups. Among patients, there was no significant difference in weight or BMI between male and female patients. However, male patients were significantly taller than female patients ($p<0.001$) and were also slightly older ($p>0.05$).

In terms of BIA values at 50 kHz, female volunteers had significantly higher impedance, resistance, and reactance values compared to male volunteers ($p<0.01$). The phase angle and capacitance did not differ significantly between the two groups. In the patient group, however, there were no significant differences in any of the BIA parameters.

ESM Table S5 also describes various body fluid composition measures comparing female and male volunteers and patients. In terms of dry weight, female volunteers had a significantly lower value compared to male volunteers. However, there was no significant difference in VE or FO between female and male volunteers. When observing patients, females had a significantly lower TBW and TBW% compared to male patients. The same pattern was observed for ECW and ICW, where female patients had significantly lower values compared to male patients ($p<0.01$). However, there was no significant difference in the ECW/ICW ratio or ECW% and ICW% between female and male patients (ESM Figure 1).

Regarding the distribution of body fluids, female volunteers had significantly lower ECS, ECF, and plasma fluid volumes compared to male volunteers. Similarly, female patients had significantly lower ECS, ECF, and interstitial fluid volumes compared to male patients. There was no significant difference in FFMH% between female and male volunteers or patients. Finally, both female volunteers and patients had a significantly lower body density compared to male volunteers and patients, respectively.

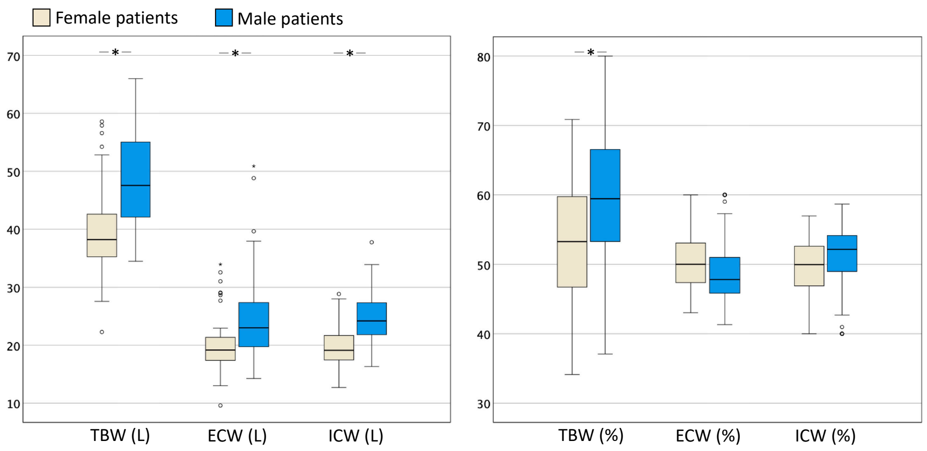

ESM Figure S1. Boxplots comparing body water distribution in female and male patients. Box and whisker plots comparing TBW, ECW and ICW expressed in absolute values (left panel), Litres (L), and relatively (right panel) as a percentage of body weight in male (n=66) and female (n=35) ICU patients. The error bars are the 95% confidence interval, the bottom and top of the box are the 25th and 75th percentiles, the line inside the box is the 50th percentile (median), and any outliers are shown as open circles. A p-value < 0.05 between groups is indicated with an *. *ECW – extracellular water; ICW – intracellular water; TBW – total body water.*

ESM Table S5. Comparison of demographic and bioelectrical impedance analysis (BIA) variables between female and male volunteers and patients. The p-values indicate whether the differences between the groups are statistically significant or not.

Variable	Female volunteers (n=68)	Male volunteers (n=33)	*p*-value	Female patients (n=66)	Male patients (n=35)	*p*-value
**Patient demographics**						
Height (cm)	169.3 ± 8.1	181 ± 6.6	0.000	163.7 ± 5.5	175.5 ± 8.3	0.000
Weight (kg)	67.4 ± 12.4	83.1 ± 14.3	0.000	77.4 ± 20.4	84 ± 20.4	NS
BMI (kg m^{-2})	23.4 ± 3.3	25.3 ± 4	0.011	28.9 ± 7.3	27.3 ± 6.7	NS
Age (years)	37.6 ± 12.6	38.9 ± 12.5	NS	60.3 ± 16.9	65.2 ± 14.8	NS
**Raw BIA values at 50 kHz**						
Impedance (Ohm)	586.8 ± 105.3	506.9 ± 62.6	0.000	404.7 ± 113.7	381.7 ± 96.4	NS
Phase angle	9.3 ± 1.5	9.5 ± 1	NS	7.9 ± 1.5	8.6 ± 3.8	NS
Resistance (Ohm)	579.5 ± 104.2	500.5 ± 61.6	0.000	401.3 ± 112.8	377.3 ± 96.1	NS
Reactance (Ohm)	94.2 ± 20.6	83.6 ± 14.2	0.009	55.1 ± 17.9	56.4 ± 24.7	NS
Capacitance (Ohm)	35.7 ± 11.3	39 ± 6	NS	63.6 ± 20.4	65.4 ± 25.2	NS
**Body fluid composition**						
Dry Weight (kg)	67.8 ± 13.4	83.1 ± 14.3	0.000	72.4 ± 18.7	78.1 ± 18.9	NS
Volume excess (L)	-0.2 ± 0.7	0 ± 1	NS	5 ± 5.2	5.9 ± 6.6	NS
Fluid Overload (%)	-0.4 ± 1.2	0 ± 1.2	NS	6.2 ± 5.8	6.9 ± 7	NS
TBW (L)	35 ± 6.7	45.7 ± 5	0.000	40.2 ± 8.6	49.6 ± 10.3	0.000
TBW (%)	52 ± 5.5	55.7 ± 5.7	0.002	53.1 ± 9.2	60.2 ± 9.3	0.000
ECW (L)	15.2 ± 3.7	19.8 ± 2.5	0.000	20.5 ± 5.7	24.6 ± 7	0.004
ECW (%)	43.2 ± 2.7	43.2 ± 1.7	NS	50.6 ± 4.3	49.1 ± 4.8	NS
ICW (L)	19.8 ± 3.4	25.9 ± 2.8	0.000	19.7 ± 3.6	25 ± 4.2	0.000
ICW (%)	56.8 ± 2.7	56.8 ± 1.7	NS	49.4 ± 4.3	50.9 ± 4.8	NS
ECW/ICW	0.8 ± 0.1	0.8 ± 0.1	NS	1.04 ± 0.19	0.98 ± 0.21	NS
ECS (L)	5.2 ± 0.9	6.8 ± 0.7	0.000	4.9 ± 0.8	6.3 ± 1	0.000
ECF (L)	15.8 ± 3.8	20.6 ± 2.6	0.000	21.4 ± 5.9	25.6 ± 7.3	0.004
Plasma Fluid (L)	3 ± 0.8	4 ± 0.6	0.000	4 ± 1.2	4.7 ± 1.4	0.016
Interstitial Fluid (L)	11.3 ± 2.7	14.5 ± 1.8	0.000	15.3 ± 4.2	18.5 ± 5.4	0.003
FFMH (%)	72.7 ± 2.4	72.5 ± 2.1	NS	79 ± 4.3	78.1 ± 4.3	NS
Body Density (Kg/L)	1.035 ± 0.014	1.046 ± 0.016	0.000	1.025 ± 0.021	1.047 ± 0.02	0.000
**Nutritional status**						
Phase angle	9.3 ± 1.5	9.5 ± 1	NS	7.9 ± 1.5	8.6 ± 3.8	NS
Malnutrition Index	0.7 ± 0.1	0.8 ± 0.1	0.051	0.89 ± 0.14	0.9 ± 0.18	NS
RMR (kcal)	1571.2 ± 205.9	1971.5 ± 202.2	0.000	1445.8 ± 186.4	1753 ± 282.4	0.000
FFM (kg)	48.1 ± 8.5	63 ± 6.3	0.000	50.6 ± 9	63.4 ± 11.3	0.000
FFM (%)	71.5 ± 6.5	76.8 ± 7.4	0.000	67.1 ± 10.2	76.9 ± 8.9	0.000
Fat (kg)	19.8 ± 7.4	20.1 ± 10.5	NS	26.8 ± 15.4	20.6 ± 12.2	0.029
Fat (%)	28.5 ± 6.5	23.2 ± 7.4	0.000	32.9 ± 10.2	23.1 ± 8.9	0.000
BCM (kg)	28 ± 5.1	35.6 ± 4	0.000	26.9 ± 5.5	33.5 ± 6	0.000
ECM (kg)	20.1 ± 4.4	27.4 ± 3.1	0.000	23.7 ± 4.6	29.9 ± 6.9	0.000
Protein (kg)	11.2 ± 2	14.4 ± 1.5	0.000	10.9 ± 2.1	13.4 ± 2.4	0.000
Mineral (kg)	4.4 ± 0.6	5 ± 0.5	0.000	4.3 ± 0.9	4.7 ± 0.8	0.042
Muscle (kg)	24.6 ± 5.2	33 ± 3.5	0.000	22.8 ± 5.1	30 ± 5.5	0.000
TBK (g)	124.1 ± 25	169.1 ± 17.7	0.000	116.2 ± 22.9	154.7 ± 26.7	0.000
TBCa (g)	1020.5 ± 180.6	1346 ± 128.2	0.000	963.6 ± 165.4	1241.9 ± 192.9	0.000
Glycogen (g)	468.5 ± 82.3	599.8 ± 63	0.000	442.7 ± 87.2	545.1 ± 94.1	0.000

BCM – body cell mass; BMI – body mass index; ECF – extracellular fluid; ECM – extracellular mass; ECS – extracellular solids; ECW – extracellular water; FAT% – fat percentage; FFM – Fat-free mass; FFMH – Fat-free mass hydration; ICW – intracellular water; RMR – resting metabolic rate; TBCa – total body calcium; TBK – total body kalium; TBW = total body water; VE = volume excess.

ESM Table S5 also compares various nutritional parameters between female and male volunteers and patients. In terms of phase angle, there was no significant difference between females and males among both volunteers and patients. However, the

malnutrition index was significantly lower in female volunteers compared to male volunteers (p=0.05), but there was no significant difference between female and male patients. Resting metabolic rate (RMR), fat-free mass (FFM), FFM percentage, body cell mass (BCM), extracellular mass (ECM), protein, mineral, muscle, total body potassium (TBK), total body calcium (TBCa), and glycogen values were all significantly higher in male volunteers compared to female volunteers. Similarly, all these parameters were also significantly higher in male patients compared to female patients. There was no significant difference in fat mass (kg) between female and male volunteers, but fat mass (in kg and %) was significantly higher in female patients compared to male patients.

Differences Between Males and Females

Next, we examined the gender differences in BIA-derived parameters. We found evidence of a gender-specific divergence in certain parameters, with a discrepancy in ECW, ICW, and TBW between males and females. Specifically, males tended to have higher water composition, as also demonstrated by a study conducted by Ritz et al. [1]. On the other hand, females had a significantly higher fat mass percentage (FAT%) and therefore a lower FFM and FFM% compared to males [2]. As fat mass contains less water than fat-free mass, it is reasonable to conclude that males generally have more body water than females. Interestingly, gender-independent metrics, such as ECW%, ICW%, and the ECW/ICW ratio, were comparable among both male and female subjects. This suggests that these measures may be more reliable in assessing fluid status in both genders, regardless of body composition differences.

We also observed that males had a significantly higher resting metabolic rate and body cell mass than females, which may reflect the impact of gender on metabolic processes. However, there was no significant difference in the malnutrition index between male and female patients, indicating that both genders are equally susceptible to malnutrition in the ICU setting.

ESM Table S6: Demographics, laboratory results, and raw BIA parameters in hospital survivors vs. non-survivors

	Total	Alive (n=53)	Died (n=48)	p Value
**Patient demographics**				
Male/female	2/1	2/1	2/1	NS
Hospital stay (days)	51.9 ± 47.5	60 ± 49	42.9 ± 44.6	0.071
ICU stay (days)	31.2 ± 26.7	33.6 ± 29.6	28.5 ± 22.9	NS
Day measurement	4.8 ± 2.1	4.8 ± 2.1	4.9 ± 2.2	NS
Height (cm)	171.4 ± 9.4	172.6 ± 8.7	170.1 ± 9.9	NS
Weight (kg)	81.7 ± 20.6	82.5 ± 21	80.8 ± 20.3	NS
BMI (kg/m^2)	27.8 ± 6.9	27.7 ± 6.6	28 ± 7.3	NS
Age (years)	63.5 ± 15.7	58.6 ± 16.8	68.9 ± 12.4	0.001
APACHE	23.3 ± 9.1	21.4 ± 9	25.4 ± 8.8	0.027
SAPS	55.5 ± 18.9	49.5 ± 17	61.8 ± 18.9	0.001
SOFA	9.8 ± 4.1	8.8 ± 3.1	10.8 ± 4.7	0.018
IAP (mmHg)	13.2 ± 3.9	12.3 ± 3.9	14 ± 3.8	0.053
EVLWI (ml/kgPBW)	10.6 ± 3.2	10.6 ± 2.7	10.6 ± 3.7	NS
**Laboratory results**				
Hematocrit (%)	28.9 ± 5.8	29 ± 5.9	28.7 ± 5.7	NS
Total protein (g/L)	49.6 ± 8.4	48.8 ± 8.6	50.5 ± 8.2	NS
Albumin (g/L)	24.5 ± 5.2	24.7 ± 6.5	24.2 ± 3.6	NS
CRP (mg/dL)	164.2 ± 113.6	171.1 ± 114.3	156.6 ± 113.6	NS
Urea (mg/DL)	69.6 ± 45.9	63.7 ± 46.2	76 ± 45.1	NS
Osmol (measured)	298 ± 18	297.9 ± 20.7	298.1 ± 14.1	NS
Osmol (calculated)	314.4 ± 40.4	315.7 ± 26.7	312.9 ± 51.5	NS
Glucose (mg/dL)	142.8 ± 52.2	143.7 ± 54.7	141.8 ± 49.8	NS
Na (mmol/L)	142.4 ± 8	142.5 ± 9	142.3 ± 6.8	NS
K (mmol/L)	4.2 ± 0.6	4.2 ± 0.6	4.1 ± 0.6	NS
Creatine (mg/dL)	1.3 ± 1	1.1 ± 0.7	1.6 ± 1.2	0.003
CCR (mL/min)	99.5 ± 48.8	114.9 ± 49.1	82.5 ± 42.7	0.001
GFR (mL/min)	82.6 ± 45.4	96.8 ± 45.5	66.9 ± 40.1	0.001
**Raw BIA values at 50 kHz**				
Impedance (Ohm)	389.7 ± 102.8	406.5 ± 101.2	371.1 ± 102.3	0.084
Phase angle	8.3 ± 3.2	8.8 ± 4	7.8 ± 2	0.098
Resistance (Ohm)	385.6 ± 102.2	401.5 ± 101.4	368.1 ± 101.4	NS
Reactance (Ohm)	55.9 ± 22.5	61.6 ± 24.7	49.7 ± 18	0.007
Capacitance (Ohm)	64.8 ± 23.5	58.3 ± 20.9	71.9 ± 24.4	0.003

ESM Table S7: Body fluid composition and nutritional status in hospital survivors vs. non-survivors

	Total	Alive (n=53)	Died (n=48)	p Value
**Body fluid composition**				
Dry Weight (kg)	76.1 ± 19	77.8 ± 18.2	74.3 ± 19.8	NS
Cumulative FB (L)	7.1 ± 6.2	6.6 ± 5.9	7.7 ± 6.6	NS
Volume excess (L)	5.6 ± 6.1	4.7 ± 6.4	6.5 ± 5.7	NS
Fluid Overload (%)	6.7 ± 6.6	5.2 ± 5.6	8.2 ± 7.2	0.022
TBW (L)	46.3 ± 10.7	46.7 ± 11.6	46 ± 9.7	NS
TBW (%)	57.8 ± 9.8	57.4 ± 9.7	58.1 ± 10	NS
ECW (L)	23.2 ± 6.8	22.9 ± 7.5	23.5 ± 6.1	NS
ECW (%)	49.6 ± 4.7	48.4 ± 4.2	50.9 ± 4.9	0.007
ICW (L)	23.1 ± 4.8	23.8 ± 4.8	22.4 ± 4.6	NS
ICW (%)	50.4 ± 4.7	51.6 ± 4.2	49.1 ± 4.9	0.007
ECW/ICW	1 ± 0.2	0.95 ± 0.18	1.06 ± 0.22	0.009
ECS (L)	5.8 ± 1.2	6 ± 1.2	5.6 ± 1	0.070
ECF (L)	24.1 ± 7.1	23.8 ± 7.8	24.5 ± 6.3	NS
Plasma Fluid (L)	4.5 ± 1.4	4.5 ± 1.6	4.5 ± 1.2	NS
Interstitial Fluid (L)	17.4 ± 5.2	17 ± 5.6	17.7 ± 4.8	NS
FFMH (%)	78.4 ± 4.3	77.7 ± 4	79.1 ± 4.5	NS
Body Density (Kg/L)	1.039 ± 0.023	1.04 ± 0.023	1.039 ± 0.022	NS
**Nutritional status**				
Phase angle	8.3 ± 3.2	8.8 ± 4	7.8 ± 2	0.098
Malnutrition Index	0.9 ± 0.16	0.86 ± 0.16	0.94 ± 0.16	0.024
RMR (kcal)	1646.5 ± 291.9	1702.6 ± 312.4	1584.7 ± 256.7	0.042
FFM (kg)	58.9 ± 12.2	59.8 ± 13.1	58 ± 11.2	NS
FFM (%)	73.5 ± 10.4	73.7 ± 10.6	73.2 ± 10.3	NS
Fat (kg)	22.8 ± 13.7	22.7 ± 13.3	22.8 ± 14.1	NS
Fat (%)	26.5 ± 10.4	26.3 ± 10.6	26.8 ± 10.3	NS
BCM (kg)	31.2 ± 6.6	32.1 ± 6.5	30.1 ± 6.7	NS
ECM (kg)	27.7 ± 6.8	27.7 ± 7.8	27.8 ± 5.8	NS
Protein (kg)	12.5 ± 2.6	12.9 ± 2.6	12.1 ± 2.5	NS
Mineral (kg)	4.6 ± 0.9	4.7 ± 0.9	4.4 ± 0.8	0.081
Muscle (kg)	27.5 ± 6.3	28.6 ± 6.5	26.3 ± 6	0.065
TBK (g)	141.4 ± 31.3	146.7 ± 33	135.5 ± 28.5	0.070
TBCa (g)	1145.4 ± 226.3	1184.2 ± 238.3	1102.7 ± 206.3	0.071
Glycogen (g)	509.6 ± 103.6	529.2 ± 108.5	488 ± 94.5	0.046

References

1. Ritz P, Vol S, Berrut G, Tack I, Arnaud MJ, Tichet J: **Influence of gender and body composition on hydration and body water spaces**. *Clin Nutr* 2008, **27**(5):740-746.
2. Malczyk E, Dziegielewska-Gesiak S, Fatyga E, Ziolko E, Kokot T, Muc-Wierzgon M: **Body composition in healthy older persons: role of the ratio of extracellular/total body water**. *J Biol Regul Homeost Agents* 2016, **30**(3):767-772.
